# Impact of COPD or Asthma on the Risk of Atrial Fibrillation: A Systematic Review and Meta-Analysis

**DOI:** 10.3389/fcvm.2022.872446

**Published:** 2022-04-11

**Authors:** Zhengbiao Xue, Siyu Guo, Xiao Liu, Jianyong Ma, Wengen Zhu, Yue Zhou, Fuwei Liu, Jun Luo

**Affiliations:** ^1^Department of Critial Care Medicine, The First Affiliated Hosptial of Gannan Medical University, Ganzhou, China; ^2^Department of Cardiology, The Affiliated Ganzhou Hospital of Nanchang University, Guangzhou, China; ^3^Medical Department, Queen Mary school, Nanchang University, Nanchang, China; ^4^Department of Cardiology, Sun Yat-sen Memorial Hospital of Sun Yat-sen University, Guangzhou, China; ^5^Department of Pharmacology and Systems Physiology, University of Cincinnati College of Medicine, Cincinnati, OH, United States; ^6^Department of Cardiology, The First Affiliated Hospital of Sun Yat-Sen University, Ganzhou, China; ^7^State Key Laboratory of Ophthalmology, Zhongshan Ophthalmic Center, Sun Yat-sen University, Guangzhou, China

**Keywords:** chronic obstructive pulmonary disease, asthma, atrial fibrillation, risk factor, meta-analysis

## Abstract

**Background:**

Respiratory diseases related to chronic pulmonary ventilation dysfunction are mainly composed of chronic obstructive pulmonary disease (COPD) and asthma. Our meta-analysis aimed to illustrate the association of COPD or asthma with risk of atrial fibrillation (AF).

**Methods:**

We systematically searched the databases of the PubMed, Embase, and Cochrane library until December 2021 for studies focusing on the relationship between COPD or asthma and AF risk. Due to the potential heterogeneity across studies, the random-effects model was used to pool the studies.

**Results:**

Our meta-analysis included 14 studies. Based on the random-effects model, the pooled analysis showed that COPD (risk ratio[RR] = 1.74, 95% confidence interval [CI]: 1.70–1.79) and asthma (RR = 1.08, 95% CI: 1.04–1.12) were significantly associated with an increased risk of AF. The results did not change after each study was excluded.

**Conclusion:**

Our current data suggested that COPD or asthma with associated with an increased risk of AF.

## Introduction

Chronic obstructive pulmonary disease (COPD) is characterized by irreversible obstruction and abnormal inflammation in the airways, and progressively or partially leads to pulmonary heart disease and respiratory failure (1). As the third leading death factor for worldwide people in 2016, COPD and its following manifestations induce serious social and economic burdens for health organizations (2). Meanwhile, asthma is another heterogeneous pulmonary disease composed of chronic airways inflammation and airway hyperresponsiveness, progressively inducing airway narrowing and remodeling with disease development (3). Following the progression of these pulmonary obstructive diseases, the presence of associated complications becomes the leading threat for patients' survival probability and quality of life (4). Therefore, the management of these complications serves as a crucial step for COPD or asthma treatment (4). Among these complications, adverse cardiovascular events (e.g., atrial fibrillation [AF], ischaemic heart disease, cerebrovascular disease, heart failure, arrhythmia, and hypertension) are regarded as one of the commonest factors for COPD and asthma patients' disease development, survival rate, and medical resource consumption (5–8). However, the relationship between cardiovascular complications and pulmonary ventilation dysfunction diseases is not well understood in clinical situations.

Romiti et al. (9) reported that COPD was prevalent in 13% of patients with AF, and was associated with increased adverse effects, the burden of management, and poorer prognosis, with double times the possibility of all-cause death and death of cardiovascular diseases and major bleeding. Based on the study of Huang Q et al. (10) various characteristics serve as the risk factors for AF development, such as older age, males, and white race. In the study of Lee et al. (7), the progression and recurrence of AF were independently influenced by COPD, and the outcomes of COPD patients with AF were worsened than those of people with sinus rhythm. Herein, our current study aimed to illustrate the association of COPD or asthma with the risk of AF.

## Methods

### Search Strategy

We systematically searched the PubMed, Embase, and Cochrane library until December 2021 for all the potential studies focusing on the relationship between COPD or asthma and AF risk. We applied the following keywords and search terms to search the relevant studies: (COPD OR chronic obstructive pulmonary disease OR asthma OR lung function OR pulmonary disease) AND (atrial fibrillation). For example, Supplementary Table 1 shows the search strategy of the PubMed database. In addition, the reference lists of relevant studies were checked to find out additional studies. In the search process of this study, we applied no language restrictions.

### Inclusion and Exclusion Criteria

Studies were included if they reported the relationship between COPD or asthma and the risk of AF after the multivariate analysis (expressed as adjusted effect estimates). The study type was not restricted in our meta-analysis. COPD, which is widely regarded as the result of chronic emphysema and bronchitis, is characterized by irreversible obstruction and inflammation in the airways. Based on the pathological physiology of asthma, reversible inflammation and airway hyperresponsiveness contribute to the progression and development of airway obstruction. The definitions of disease conditions (COPD or asthma) and outcome (AF) were applied according to the originally included studies.

We excluded studies that only reported the crude event rate of AF associated with COPD or asthma. Studies with certain study types, such as reviews, editorials, letters, commentaries, trial protocols, meta-analysis and case reports, were excluded because they had no relevant data used in the pooled analysis. In addition, we did not include the meeting abstracts due to the insufficient data for study quality assessment, and the different objectives for our study.

### Data Extraction and Quality Assessment

Two reviewers first independently screened the titles and abstracts of the records from the databases to pick up the potential studies, the full text of which were subsequently screened to identify the final studies that met the eligibility criteria. Discrepancies and disagreements were resolved by consensus. The baseline characteristics of the included studies were extracted, such as the authors, design of the study, data source, sample size, age and sex of the studied population, definitions of COPD or asthma, method of AF assessment, follow-up time, and effect estimates.

And then, the quality assessment was performed by using the Newcastle-Ottawa Scale (NOS) including three domains: the selection of cohorts (0–4 scores), the comparability of cohorts (0–2 scores), and the assessment of the outcome (0–3 scores). In this meta-analysis, the NOS of ≥ 6 and <6 scores were regarded as moderate-to-high quality and low-quality, respectively.

### Heterogeneity Test

We assessed the heterogeneity across the included studies by using the Cochrane Q test and *P* value as well as the I^2^ values. In our present meta-analysis, *P* < 0.1 and I^2^ > 50% suggested significant heterogeneity.

### Statistical Analysis

For each included study, adjusted odds ratios (ORs), risk ratios (RRs), or hazard ratios (HRs), as well as their confidence intervals (CIs), were extracted. In this study, we opt to refer to all the effect estimates as RRs, and this choice would not affect our results. The natural logarithms of the RR (Ln[RR]) and their standard errors (SELn[RR]) were calculated, and then pooled using a random-effects model weighted by the inverse-variance method. In the sensitivity analysis, we excluded one study at a time to examine the effect of each study on the pooled results. In addition, we used the fixed-effects model to re-perform the above-mentioned meta-analysis. The subgroup analyses were not performed due to the limiting studies. The publication bias was evaluated by employing the funnel plots for visual inspection of asymmetry., and was further assessed statistically by using The Egger's and Begg's tests were used for assessing the statistical publication bias, where a *P*-value of > 0.1 indicated no significant publication bias.

All the statistical analyses were performed using the Review Manager version 5.4 software (the Cochrane Collaboration 2014, Nordic Cochrane Centre Copenhagen, Denmark), and the Stata software (version 15.0, Stata Corp LP, College Station, TX). In this study, the statistical significance threshold was set at a *P*-value of < 0.05.

## Results

### Study Selection

**Figure 1:**
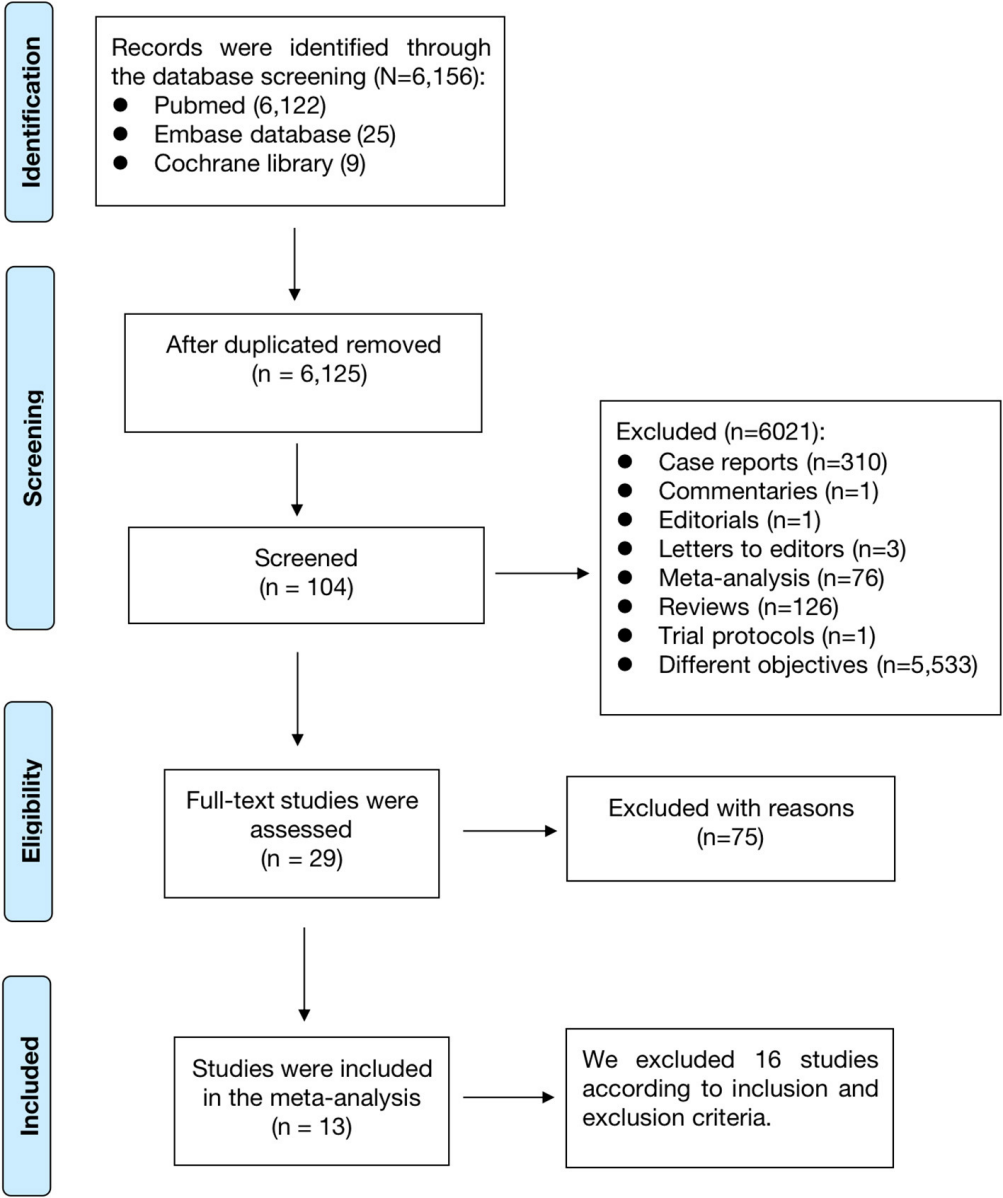
The process of the literature search of this meta-analysis.

As shown in Figure 1, a total of 6,156 records were retrieved in the PubMed, Embase databases and Cochrane library. After the first and second phases of screenings, 29 remaining studies were potentially available. Finally, a total of 13 observational studies were included in this meta-analysis (11–23).

The baseline characteristics of included studies are shown in Table 1. These studies were published from 2005 to 2020, and the sample size ranged from 4,267 to 28,000,000 participants. All these 13 studies covered every age group of adults (≥18 years), and the follow-up period lasted several years to collect sufficient information. As shown in Supplementary Table 2, all the included studies had a NOS of ≥ 6 scores.

**Table 1:** Baseline characteristics of the included studies.

							**Definitions**			**AF at**
							**of COPD**	**AF**	**Follow-up**	**baseline**
**Included studies**	**Country**	**Study type**	**Data source**	**Sample size**	**Age (y)**	**Male (%)**	**or asthma**	**assessment**	**(y)**	**(yes or no)**
Knuiman et al. (24)	Australia	Cohort study	Busselton community	4,267	25–84	43.6	Spirometry	ECG	1	No
Lip et al. (25)	Denmark	Cohort study	Danish citizens	2,499,235	70	47.1	Spirometry	ECG	14	No
Li et al. (11)	China	Derivation cohort	Yunnan medical insurance database	471,446	31–74	52.7	Spirometry	ECG	11	No
Grymonprez et al. (12)	Netherlands	Cohort study	Rotterdam study	10,943	≥45	63.1	Spirometry	ECG	2	No
Sidney et al. (13)	United States	Cohort study	Members of the KPNG	45,966	≥40	55.4	Spirometry	ECG	3	Yes
Carter et al. (14)	United Kingdom	Cohort study	All patients of 7 NHS hospitals	1,220,024	≥18	41.9	Spirometry	ECG	13	Yes
Konecny et al. (15)	United States	Cohort study	All unique adult patients of Mayo Clinic Rochester	7,441	48–80	51.0	Spirometry	ECG	9	No
Mapel et al. (16)	United States	Cohort study	All hospitalized veterans	948,633	44–76	97.7	Spirometry	ECG	6	No
Li et al. (17)	France	Cohort study	Entire population of France covering hospital care	240,459	≥18	52.4	Spirometry	ECG	4	No
Tattersall et al. (18)	United States	Cohort study	Participants free of known CVD	6,814	Mean 62.0	47.0	Spirometry	ECG	2	No
Chan et al. (19)	Taiwan	Nested case-control study	National Health Insurance Research Database	17,514	≥18	47.0	Spirometry	ECG	7	Yes
Cepelis et al. (20)	Norway	Cohort Study	Second and third iteration of the survey-based Nord-Trondelag Health Study (HUNT)	54,567	≥20	47.2	Spirometry	ECG	0.5	No
Li et al. (21)	China	Cohort Study	PUTH electronic medical database	23,523	>18	46.1	Spirometry	ECG	20	No

*COPD, chronic obstructive pulmonary disease; AF, atrial fibrillation; KPNG, Kaiser Permanente Medical Care Program; NHS, National Health Service; CVD, cardiovascular disease; PUTH, Peking University Third Hospital*.

### Impact of COPD on AF Risk

A total of 12 included studies assessed the association of COPD with AF risk (11–19, 23). Grymonprez et al. reported the relationship between the frequency of COPD and AF incidence, and also the association of different systemic inflammatory levels and AF risk. Knuiman et al. reported the race- and sex-specific association of COPD with AF incidence, separately. We combined the separate data of these 2 studies as the final effect estimates in the pooled analysis.

**Figure 2:**
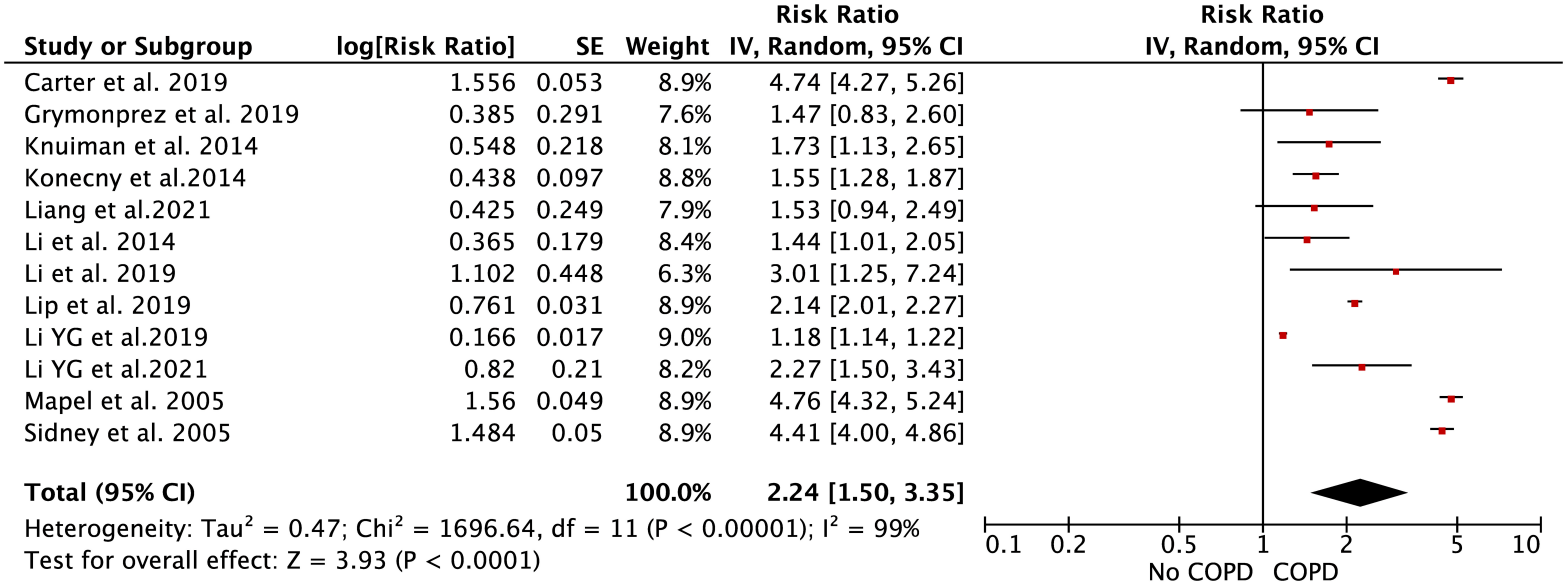
Forest plot for the relationship between chronic obstructive pulmonary disease and the development of atrial fibrillation.

In the pooled analysis using the random-effects model, the effect estimate of COPD on AF risk was 2.24 (RR = 2.24, 95% CI: 1.47–3.41; Figure 2). In the sensitivity analysis, the pooled results were stable after exclusion one study at a time. In addition, the pooled RRs were not significantly changed when we re-performed the analyses using the fixed-effects model (RR = 1.74, 95% CI: 1.70–1.79; Supplementary Figure 1).

### Impact of Asthma on AF Risk

A total of 5 included studies focused on the relationship between asthma and AF risk (16, 20–22). Li et al. (13) reported the race- and sex-specific association of asthma with AF incidence, separately. Tattersall et al. (20) reported the association of intermittent or persistent asthma with AF risk, separately. We combined the separate data of these 2 studies as the final effect estimates in the pooled analysis.

**Figure 3:**
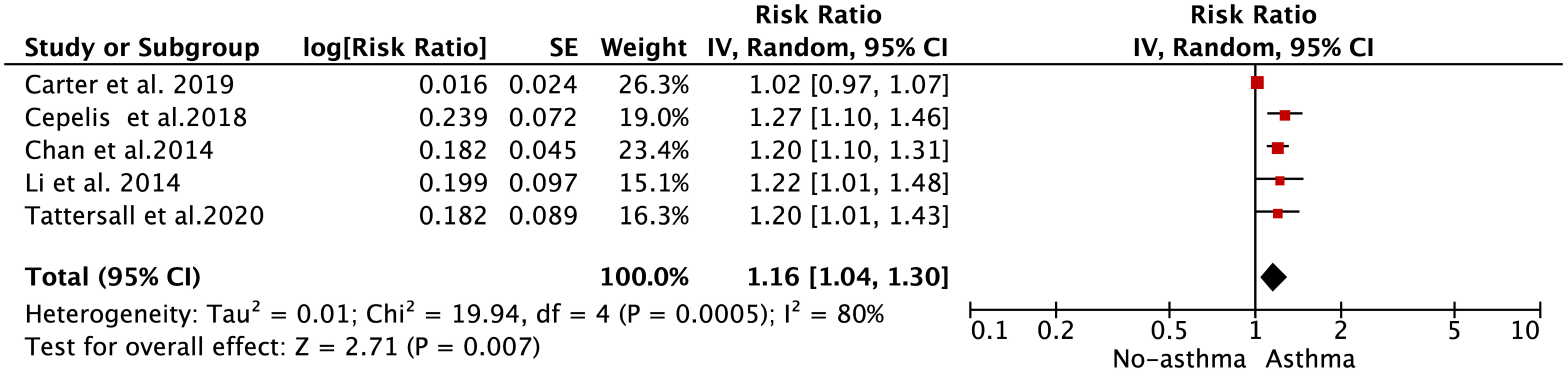
Forest plot for the relationship between asthma and the development of atrial fibrillation.

As shown in Figure 3, the pooled result from the random-effects model showed that asthma was associated with an increased risk of AF (RR = 1.16, 95% CI: 1.04–1.30). In the sensitivity analysis, the pooled results were stable after exclusion one study at a time. In addition, the results from the fixed-effects model (RR = 1.08, 95% CI: 1.04–1.12; Supplementary Figure 2) were similar to those from the random-effects model.

### Publication Bias

When assessing the effect of COPD on AF risk, the funnel plot presented in Supplementary Figure 3 suggested no potential publication bias. The Egger's (*P* = 0.199; Supplementary Figure 4) and Begg's (*P* = 0.631) tests also showed no significant publication bias. For the section on asthma and AF, it was unsuitable to assess the publication bias because <10 studies were included for the quantitative analysis according to the Cochrane handbook.

## Discussion

The leading advantage of our study was the first meta-analysis to identify the association between COPD or asthma and AF risk. Especially, we quantified the correlation between the magnitude of AF and chronic pulmonary ventilation diseases (COPD and asthma). The pooled results demonstrated that the increased incidence of AF was associated with COPD or asthma. There was no variance in results when one study was excluded at a time.

Several previous studies have revealed that the increased risk of AF was commonly observed in patients with COPD or asthma. Yucel et al. reported that the biomarker cancer antigen 125 (CA-125) reached a higher level in patients with cardiopulmonary diseases, such as AF and COPD (26). This study also revealed that the development of AF was highly correlated to CA-125 elevation and COPD progression (26). Simons et al. (27) reported that exception of other common risk factors, the exacerbation of COPD served as an independent risk factor for AF in patients with COPD (27). Moreover, other associated studies also reported that oxidative stress response, inflammation, the overdose of β2-agonists, and autonomic changes were potential molecular pathways to promote AF development in patients with COPD or asthma (28–31). However, there is insufficient convincing evidence to formally quantify the increased risk of AF in patients with COPD or asthma. By summarizing relevant studies, we clarified the relationship between COPD or asthma and the risk of AF and concluded that COPD and asthma were related to the risk of AF.

Recent studies have demonstrated the potential pathophysiological mechanisms which induce AF in patients with COPD or asthma. Among these pathological manifestations, hypoxia plays a significant role in oxidative stress response, which contributes to the abnormal electrophysiological change of atrial muscle cells and finally leads to AF (30, 31). Yang et al. (32) have reported that chronic intermittent hypoxia promotes atrial remodeling to alter the atrial autonomic structures, then increases AF inducibility. Based on the pathophysiological mechanism of COPD and chronic intermittent asthma, both hypercapnia and hypoxemia caused by COPD can lead to contraction of pulmonary arterioles, thus increasing the pressure of the pulmonary artery and the right atrium. The elevation of right atrial pressure will cause the expansion of the right atrium and the hemodynamic changes of endocardial vessels, resulting in atrial contraction, blood flow reset, and increase susceptibility to arrhythmia, such as AF (33). Based on some recent studies, due to the distribution of β2 receptors on the cardiac muscle, inhalation of β2-agonists in COPD and asthma patients becomes another potential predisposing factor to trigger the alteration of cardiac electrophysiology, which includes AF (28–31).

By analyzing our results of this meta-analysis, it showed that the presence of AF was more common in COPD and asthma patients. Grymonprez M et al. (12) and Konecny T et al. (15) revealed that frequent exacerbation and severity of COPD were positively correlated to the incidence of AF, and COPD patients also suffered from different levels of enlarged left atrium and systemic inflammatory response. Moreover, the abnormal dilation of the ventricle and atrium leads to cardiac muscular damage and systolic dysfunction, which also contribute to AF occurrence (34, 35).

Understanding the relationship between chronic pulmonary ventilation dysfunction diseases and cardiovascular complications, such as AF, plays an important role in disease management. For example, some targeted treatments and medication usages will be emphasized in the novel handbook of COPD (and asthma) management. Some independent studies have substantially identified the increased cardiovascular risks in patients with COPD and asthma, and some associated evidence was provided by retrievable researches and reviews (9, 10). However, no previous study provided enough quantitative synthesis evidence to identify the magnitude of the increased AF risk among patients with COPD or asthma. In our meta-analysis, we have built up the relationship between COPD or asthma and AF risk and provided a future researching direction, which includes the identification of basic molecular mechanisms of COPD and asthma leading to AF.

### Limitations

Our current meta-analysis had several limitations. First, although the observed results were significant based on the statistical aspects, the heterogeneity was still evident across the included studies. Potential causes included inconsistent definitions of COPD or asthma, different study types, and various studied populations. Further prospective cohort studies containing larger sample size, longer-period follow-up, and more subgroups analysis could confirm our findings. Second, we were unable to perform the subgroup analysis based on the types of AF because the originally included studies did not report them separately. Third, due to the inadequate raw data and confounding factors (e.g., race, duration of follow-up, number of cases.), we did not perform the subgroup analyses. Finally, among the patients with COPD, the association between the frequency and severity of COPD and AF incidence was observed in some related studies. However, due to the limited number of these studies, our meta-analysis was not able to evaluate this situation.

## Conclusion

Currently available data suggested an association of COPD or asthma with the increased AF risk. Further high-quality studies should be conducted to confirm the relationship between COPD or asthma and AF risk.

## Data Availability Statement

The original contributions presented in the study are included in the article/Supplementary Material, further inquiries can be directed to the corresponding authors.

## Author Contributions

All authors listed have made a substantial, direct, and intellectual contribution to the work and approved it for publication.

## Funding

This study was funded by National Natural Science Foundation of China (82100273 and 21866019).

## Conflict of Interest

The authors declare that the research was conducted in the absence of any commercial or financial relationships that could be construed as a potential conflict of interest.

## Publisher's Note

All claims expressed in this article are solely those of the authors and do not necessarily represent those of their affiliated organizations, or those of the publisher, the editors and the reviewers. Any product that may be evaluated in this article, or claim that may be made by its manufacturer, is not guaranteed or endorsed by the publisher.
