# Supplementary material for: Impact of COPD or Asthma on the Risk of Atrial Fibrillation: A Systematic Review and Meta-Analysis
Source: Front Cardiovasc Med. 2022 Apr 11;9:872446. doi: 10.3389/fcvm.2022.872446 (PMC9035743; doi:10.3389/fcvm.2022.872446)
Supplement: Supplementary file 1 [file Data_Sheet_1.PDF]

Supplementary Table 1. Retrieval strategy in the Pubmed database

Search number	Query	Results
1	COPD	97,359
2	chronic obstructive pulmonary disease	89,748
3	asthma	203,581
4	lung function	340,944
5	pulmonary disease	1,233,863
6	1 or 2 or 3 or 4 or 5	1,485,572
7	atrial fibrillation	95,210
8	6 and 7	6,122

Supplementary Table 2. Quality assessment of the included studies based on the NOS tool

Studies	Selection				Comparability	Outcome			Total
	Exposed cohort	Non-exposed cohort	Ascertainment of exposure	Outcome of interest		Assessment of outcome	Length of follow-up	Adequacy of follow up	
Knuiman et al. 2014	*	*	*		**	*	*	*	8
Lip et al. 2019	*	*	*		**	*	*	*	8
Li et al. 2019	*	*	*		**	*	*	*	8
Grymonprez et al. 2019	*	*	*		**	*		*	7
Sidney et al. 2005	*	*	*		**	*		*	7
Carter et al. 2019	*	*	*		**	*	*	*	8
Konecny et al.2014	*	*	*		**	*	*	*	8
Mapel et al. 2005	*	*	*		**	*	*	*	8
Li YG et al.2019	*	*	*		**	*		*	7
Tattersall et al.2020	*	*	*		**	*		*	7
Chan et al.2014	*	*	*		**	*	*	*	8
Cepelis et al.2018	*	*	*		**	*		*	7
Li YG et al.2021	*	*	*		**	*	*	*	8

Study or Subgroup	log[Risk Ratio]	SE	Weight	Risk Ratio IV, Fixed, 95% CI
Carter et al. 2019	1.556	0.053	6.0%	4.74 [4.27, 5.26]
Grymonprez et al. 2019	0.385	0.291	0.2%	1.47 [0.83, 2.60]
Knuiman et al. 2014	0.548	0.218	0.4%	1.73 [1.13, 2.65]
Konecny et al.2014	0.438	0.097	1.8%	1.55 [1.28, 1.87]
Liang et al.2021	0.425	0.249	0.3%	1.53 [0.94, 2.49]
Li et al. 2014	0.365	0.179	0.5%	1.44 [1.01, 2.05]
Li et al. 2019	1.102	0.448	0.1%	3.01 [1.25, 7.24]
Lip et al. 2019	0.761	0.031	17.7%	2.14 [2.01, 2.27]
Li YG et al.2019	0.166	0.017	58.8%	1.18 [1.14, 1.22]
Li YG et al.2021	0.82	0.21	0.4%	2.27 [1.50, 3.43]
Mapel et al. 2005	1.56	0.049	7.1%	4.76 [4.32, 5.24]
Sidney et al. 2005	1.484	0.05	6.8%	4.41 [4.00, 4.86]
**Total (95% CI)**			**100.0%**	**1.74 [1.70, 1.79]**

Heterogeneity: $Chi^2 = 1696.64$, $df = 11$ ($P < 0.00001$); $I^2 = 99\%$

Test for overall effect: $Z = 42.60$ ($P < 0.00001$)

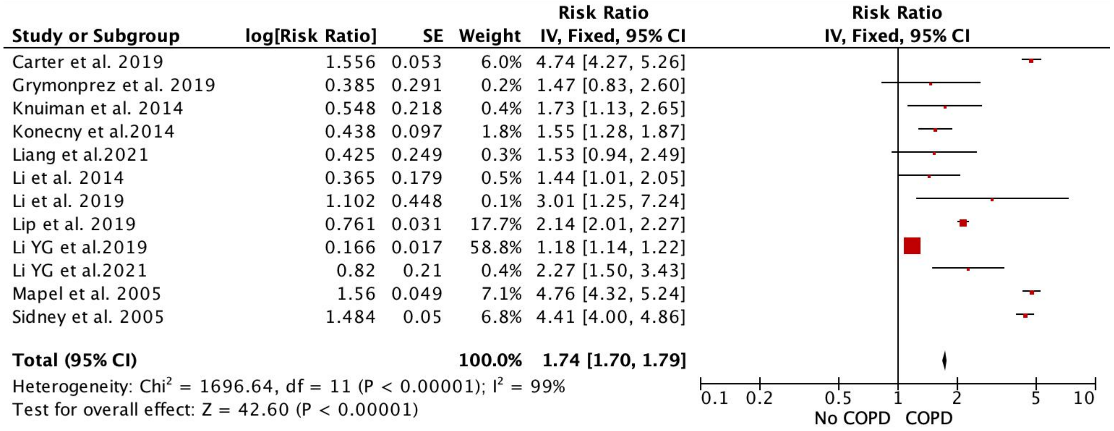

Supplementary Figure 1. Forest plot for the relationship between chronic obstructive pulmonary disease and the development of atrial fibrillation (fixed-effects model)

Study or Subgroup	log[Risk Ratio]	SE	Weight	Risk Ratio IV, Fixed, 95% CI
Carter et al. 2019	0.016	0.024	65.4%	1.02 [0.97, 1.07]
Cepelis et al.2018	0.239	0.072	7.3%	1.27 [1.10, 1.46]
Chan et al.2014	0.182	0.045	18.6%	1.20 [1.10, 1.31]
Li et al. 2014	0.199	0.097	4.0%	1.22 [1.01, 1.48]
Tattersall et al.2020	0.182	0.089	4.8%	1.20 [1.01, 1.43]
**Total (95% CI)**			**100.0%**	**1.08 [1.04, 1.12]**

Heterogeneity: $Chi^2 = 19.94$, $df = 4$ ($P = 0.0005$); $I^2 = 80\%$

Test for overall effect: $Z = 4.03$ ($P < 0.0001$)

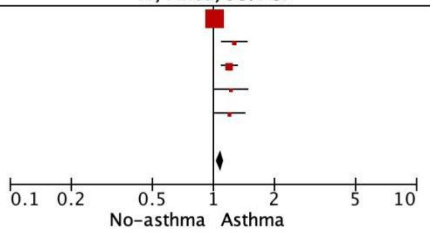

Supplementary Figure 2. Forest plot for the relationship between asthma and the development of atrial fibrillation (fixed-effects model)

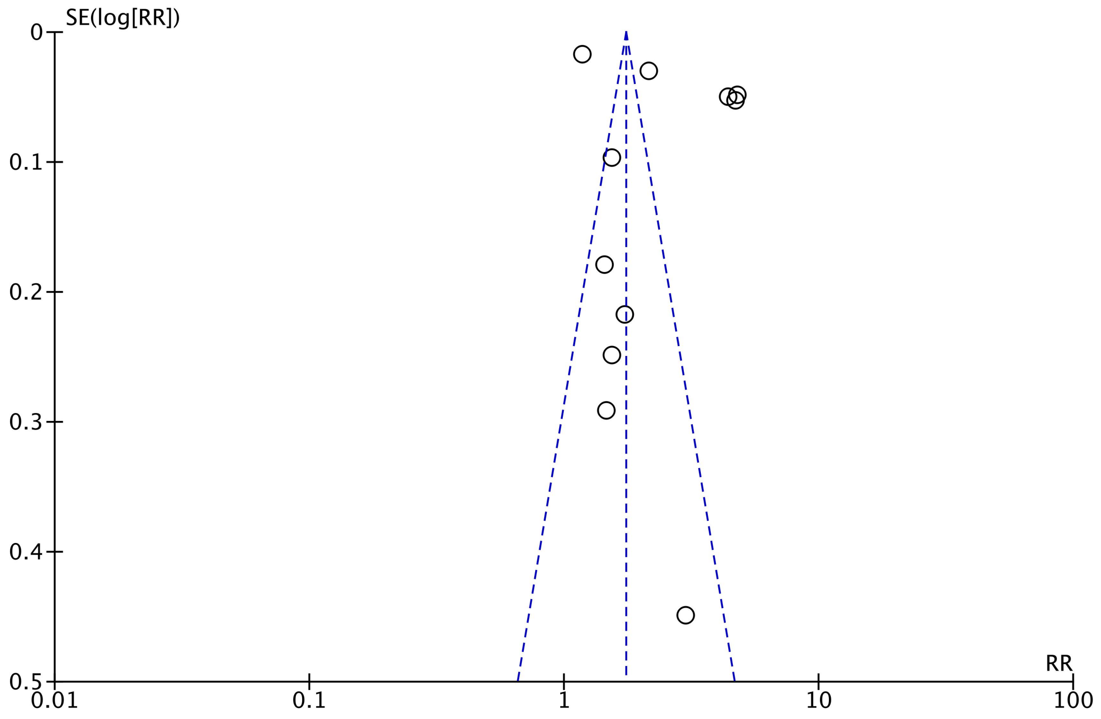

Supplementary Figure 3. Funnel plot for chronic obstructive pulmonary disease and the development of atrial fibrillation

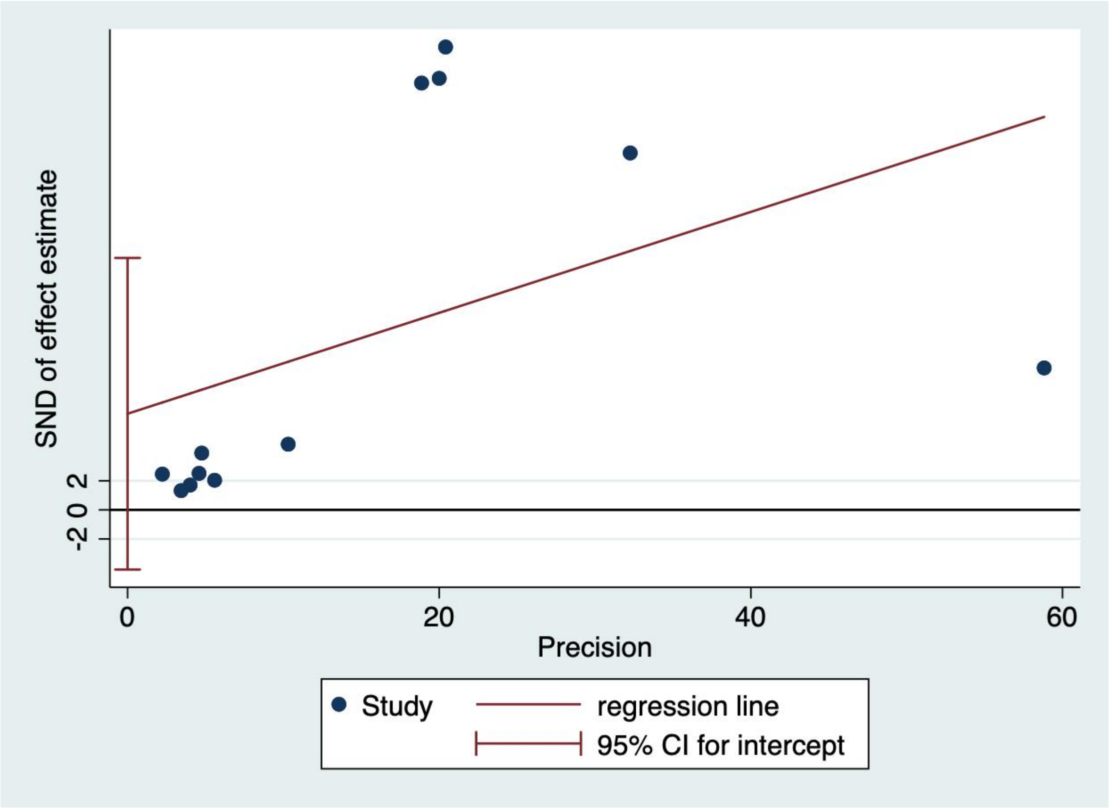

Supplementary Figure 4. The Egger's test for chronic obstructive pulmonary disease and the development of atrial fibrillation (P=0.199)
